# Continuing Professional Development

**DOI:** 10.1002/jmrs.401

**Published:** 2020-06-07

Maximise your CPD by reading the following two selected articles which appear in this issue and answer the five questions. Please remember to self‐claim your CPD and retain your supporting evidence. Answers will be available via the QR code and online at https://www.asmirt.org/news‐and‐publications/jmrs, as well as published in the subsequent JMRS issue.

## Radiation Therapy – Original Article

**Management of radiation therapy‐induced vaginal adhesions and stenosis: a New Zealand survey of current practice**

Summerfield J, Leong A. (2020) *J Med Radiat Sci.* https://doi.org/10.1002/jmrs.386

1. Radiation therapy‐induced vaginal adhesions and stenosis (RTVAS) in female patients can lead to which of the following issues?
  - Long‐term sexual dysfunction
  - Compromised pelvic examinations
  - Reduced surveillance of disease recurrence
  - All of the above
2. The Cochrane review in 2014 found studies of limited quality with conflicting outcomes regarding the effectiveness of dilators for managing RTVAS. Which of the following recommendations were made regarding vaginal dilation?
  - The use of dilators was not supported in any way
  - Use during RT, but not post‐treatment
  - Not during RT, but use post‐treatment (provided acute vaginal side effects had resolved)
  - Use during RT and post‐treatment (provided acute vaginal side effects had resolved)
3. All responding NZ departments in the RTVAS survey answered a question regarding sexual activity as a factor in the recommendation of vaginal dilator usage for female pelvic cancer patients. What was the result?
  - Dilators were recommended irrespective of sexual activity
  - Sexual activity was a contraindication for vaginal dilator usage
  - Sexual activity was a requirement for vaginal dilator usage
  - Sexual activity varied as both a contraindication and requirement for vaginal dilator usage across New Zealand
4. Lubricant is recommended to be used with dilators in the management of RTVAS. Given the long‐time frames female patients are advised to use dilators, what considerations should be made?
  - Lubricant preservatives, as these may disrupt the vaginal microbiome
  - The viscosity of the lubricant
  - Whether the lubricant is oil based or water based
  - The packaging of the lubricant
5. The NZ survey showed general agreement between RT departments for pelvic treatment sites where education is provided regarding the incidence and management of RTVAS. What were the treatment sites where dilators were recommended with 100% consensus?
  - Rectum/Anal Canal
  - Cervix/Endometrium/Vagina
  - Sarcoma/Lymphoma
  - Total Body Irradiation (TBI)

**Recommended further reading:**

1. Miles, T. and Johnson, N. Vaginal dilator therapy for women receiving pelvic radiotherapy. *Cochrane Database of Systematic Reviews*. 2014; (9): CD007291. https://www.cochranelibrary.com/cdsr/doi/10.1002/14651858.CD007291.pub3/full
2. Morris, L. Viet Do, Chard, J. Brand A. Radiation‐induced vaginal stenosis: current perspectives*.Int J Women’s Health*. 2017; **9**: 273–279. Published online. May 2, 2017. https://www.dovepress.com/radiation-induced-vaginal-stenosis-current-perspectives-peer-reviewed-article-IJWH doi: 10.2147/IJWH.S106796
3. Bakker RM, terKuile MM, Vermeer WM, et al. Sexual rehabilitation after pelvic radiotherapy and vaginal dilator use: consensus using the Delphi method*.Int J Gynecol Cancer*. 2014; 24 (8): 1499–506. https://www.semanticscholar.org/paper/Sexual-rehabilitation-after-pelvic-radiotherapy-and-Bakker-Kuile/52addbc74d69c38fac8a406258b98e4e937db76f doi: 10.1097/IGC.0000000000000253

## Medical Imaging – Original Article

**Current practice in mammographic imaging of the augmented breast in Australia**

O’Keefe JR, Wilkinson JM,Spuur KM.(2020)

*J Med Radiat Sci.* https://doi.org/10.1002/jmrs.374

1. Which of the following statements correctly explains the relationship between breast implants, cancer risk, mammography and cancer detection?
  - The reduced sensitivity of mammographic imaging due to the presence of implants leads to late cancer detection and poorer prognosis than for women with non‐augmented breasts
  - There is no conclusive evidence that the increased sensitivity of mammographic imaging due to the presence of implants leads to late cancer detection or poorer prognosis than for women with non‐augmented breasts
  - There is no conclusive evidence that the reduced sensitivity of mammographic imaging due to the presence of implants leads to late cancer detection or poorer prognosis than for women with non‐augmented breasts
  - There is conclusive evidence that the reduced sensitivity of mammographic imaging due to the presence of implants leads to late cancer detection or poorer prognosis than for women with non‐augmented breasts
2. To improve imaging of the augmented breast a dedicated method of implant imaging was devised in 1988. The Eklund technique is named after the researchers who developed the method. By what other name is the technique commonly referred?
  - Breast Displacement (BD) technique
  - Implant Displacement (ID) technique
  - Pinch Technique(PT)
  - Augmented Imaging (AI) technique
3. The ID technique has been widely adopted for routine imaging of the augmented breast. The technique is best described as displacing the implant
  - Superiorly and posteriorly toward the chest wall while the available breast tissue is pulled anteriorly and compressed
  - Superiorly and anteriorly toward the chest wall while the available breast tissue is pulled anteriorly and compressed
  - Superiorly and anteriorly toward the chest wall while the available breast tissue is pulled posteriorly and compressed
  - Posteriorly toward the chest wall while the available breast tissue is pulled and compressed
4. Which following statement is correct?
  - Implant displaced (ID) imaging should not be attempted before completion of a craniocaudal view (CC) and mediolateral oblique view (MLO) imaging series with very limited to no compression, this is in order to determine implant integrity prior to compression for ID imaging
  - Typically, ID imaging is attempted before completion of a CC and MLO imaging series with very limited to no compression, this is in order to determine implant integrity prior to compression for the ID imaging series
  - In terms of imaging technique, the order of projections including limited compression imaging is at the discretion of the radiographer
  - Non‐compressed/limited compression views should be mandatorily performed prior to ID imaging to show implant position, demonstrate the amount of tissue available for compression, assist in the planning of subsequent projections and to ensure implant integrity is sufficient prior to full compression
5. The most common complication of breast augmentation is as follows:
  - Implant rupture/leakage/deflation
  - Inflammatory reaction resulting in fibrosis and capsular contraction
  - Implant migration
  - Breast implant associated‐anaplastic large cell lymphoma (BIA‐ALCL)

## Recommended further reading:

1. Eklund GW, Busby RC, Miller SH, Job JS. Improved imaging of the augmented breast. *AMJ* [Internet]. 1988 Sep; 151(3):469–473. Available from: https://www.ajronline.org/doi/10.2214/ajr.151.3.469
2. National Health Service Breast Screening Programme. NHS Breast Screening Programme: Screening women with breast implants. Public Health England, London, 2017. Available from: https://www.gov.uk/government/publications/breast‐screening‐imaging‐women‐with‐breast‐implants
3. Robinson KA, et al. Patient‐Awareness Survey: Do Breast Implants Affect the Acquisition and Accuracy of Screening Mammography? *Journal of Breast Imaging* [Internet]. 2019 Oct; 1(4):297–302. Available from: https://academic.oup.com/jbi/article/1/4/297/5593622

## Answers to Questions Published in Previous Issue

Atkinson S, Neep M, Starkey, D. Reject rate analysis in digital radiography: an Australian emergency imaging department case study. *J Med Radiat Sci*. 2020 Mar; 67(1): 72–79. https://doi.org/10.1002/jmrs.343

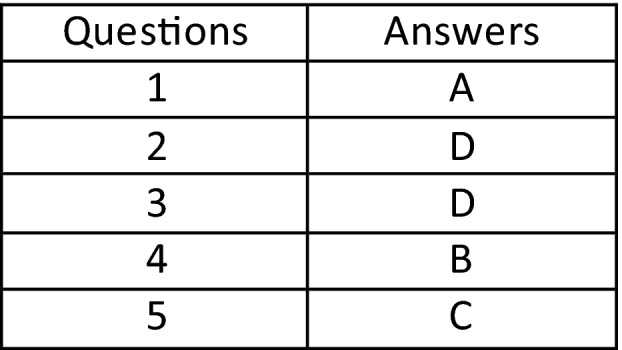

Ryan J, Willis D. Paediatric image‐guided radiation therapy: Determining and evaluating appropriate kilovoltage planar exposure factors for the Varian on‐board imager. *J Med Radiat Sci.* 2020 Mar; 67(1): 16–24. https://doi.org/10.1002/jmrs.352

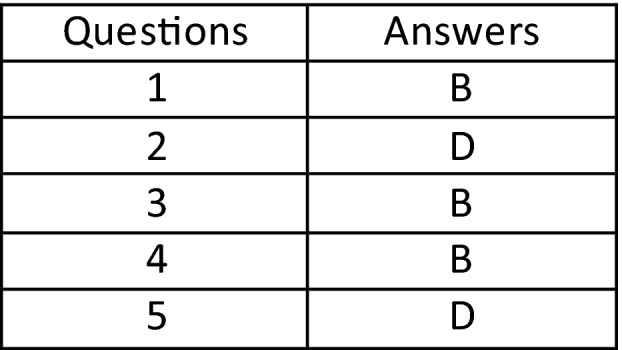

## Answers to this Issue


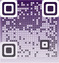


Scan this QR code to find the answers, or visit https://www.asmirt.org/news‐and‐publications/jmrs

